# ECMO use in COVID-19: lessons from past respiratory virus outbreaks—a narrative review

**DOI:** 10.1186/s13054-020-02979-3

**Published:** 2020-06-06

**Authors:** Hwa Jin Cho, Silver Heinsar, In Seok Jeong, Kiran Shekar, Gianluigi Li Bassi, Jae Seung Jung, Jacky Y. Suen, John F. Fraser

**Affiliations:** 1 Critical Care Research Group, Faculty of Medicine, University of Queensland and The Prince Charles Hospital, Brisbane, Australia; 2 Department of Pediatrics, Chonnam National University Children’s Hospital and Medical School, Gwangju, Republic of Korea; 3 Department of Thoracic and Cardiovascular Surgery, Chonnam National University Hospital and Medical School, Gwangju, Republic of Korea; 4 Adult Intensive Care Services, The Prince Charles Hospital, Brisbane, Australia; 5 ’Institut d’Investigacions Biomediques August Pi i Sunyer, Barcelona, Spain; 6 Department of Thoracic and Cardiovascular Surgery, College of Medicine, Korea University, Seoul, Republic of Korea

**Keywords:** Coronavirus disease-2019 (COVID-19), Extracorporeal membrane oxygenation (ECMO), Severe acute respiratory syndrome (SARS), Pandemic H1N1, Middle East respiratory syndrome (MERS)

## Abstract

The spread of coronavirus disease 2019 (COVID-19) continues to grow exponentially in most countries, posing an unprecedented burden on the healthcare sector and the world economy. Previous respiratory virus outbreaks, such as severe acute respiratory syndrome (SARS), pandemic H1N1 and Middle East respiratory syndrome (MERS), have provided significant insights into preparation and provision of intensive care support including extracorporeal membrane oxygenation (ECMO). Many patients have already been supported with ECMO during the current COVID-19 pandemic, and it is likely that many more may receive ECMO support, although, at this point, the role of ECMO in COVID-19-related cardiopulmonary failure is unclear. Here, we review the experience with the use of ECMO in the past respiratory virus outbreaks and discuss potential role for ECMO in COVID-19.

## Background

On December 2019, the district of Wuhan in central China announced detection of a previously undescribed virus that led to clusters of pneumonia. The disease caused by this novel severe acute respiratory syndrome coronavirus 2 (SARS-CoV-2) was subsequently named coronavirus disease 2019, the COVID-19. The SARS-CoV-2 outbreak was declared as a public health emergency of international concern by the World Health Organization (WHO) on 30 January and a pandemic on 11 March [1]. Despite lessons learnt from previous outbreaks, the preparedness and awareness for such a transmittable virus was inadequate to stop its spread of COVID-19 to over 4,700,000 patients with crude mortality of 6.6% as of May 19 2020 [2]. The mortality in mechanically ventilated COVID-19 patients remains high, and it is unclear if some of these patients may be rescued with ECMO.

**Fig. 1:**
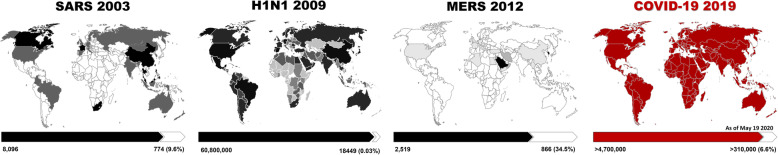
Geographical distribution in previous viral outbreaks with the name of viral outbreaks and onset year. The number of infected cases (coloured bar) and number of deaths (blank bar) with percentage of death are described under each map. The length of the bars represent the approximate ratio of infected cases to deaths. The geographic distribution in COVID-19 is not expressed in this figure since the numbers and countries are still changing. As of May 19, 2020, total infected numbers of cases are over 4,700,000 and over 310,000 (6.6%) died of COVID-19. *SARS* Severe acute respiratory syndrome; *MERS* Middle East respiratory syndrome; *COVID-19* Coronavirus disease 2019

There have been several viral outbreaks in recent memory, including severe acute respiratory syndrome (SARS), pandemic H1N1 influenza and the Middle East respiratory syndrome (MERS) (Fig. 1). Whilst the SARS outbreak in China in 2002 [3] caused an outbreak of severe acute respiratory syndrome through coronavirus (SARS-CoV) [4–8], there is minimal reported data on the use of ECMO. This was because ECMO was not commonly used at that time, even in those critically ill patients who did not respond favourably to conventional mechanical ventilation and other adjuncts [9]. There is some data on use of ECMO in MERS [10–15]. The 2009 H1N1 pandemic witnessed the rise of ECMO, and this in part can be attributed to the significant “age shift” with younger patients (< 65 years of age) getting more affected by the virus. Improvements in technology over time have certainly played a significant role too.

Since the 2009 H1N1 pandemic, more evidence has emerged support venovenous (V-V) ECMO use in ARDS [16–20]. The use of venoarterial (V-A) ECMO for cardiac support is an evolving area and certainly needs further evidence. Although ECMO has a role in selected patients in context of the current pandemic, the criteria for patient selection and timing of ECMO initiation are yet to be defined. This is important to allow judicious use of available resources such resource-consumptive circumstances [21]. In this narrative review, the focus will be on the use of ECMO during previous viral outbreak as well as in COVID-19 to learn lessons regarding guidance of treatment that will benefit all of healthcare workers and patients.

## Cardiopulmonary complications in viral outbreaks

Whilst significant pulmonary pathology is the hallmark of recent viral outbreaks which was respiratory, the incidence of significant injuries to cardiovascular system has also been reported.

Both H1N1 and MERS were associated with significant cardiopulmonary involvement. Although severe pneumonia and ARDS were mostly commonly seen complications, Dawood et al. conducted calculations of crude respiratory and cardiovascular mortality rates from H1N1, estimating the total attributable deaths at 200,000 and 80,000, respectively [22]. Fulminant myocarditis was reported during the H1N1 pandemic [23]; acute myocarditis, acute myocardial infarction, acute heart failure, pericarditis and shock were also reported in patients with MERS [13, 24–26].

In COVID-19, whilst most commonly reported pulmonary complications in critically ill patients were also pneumonia and ARDS [27–30], there are substantial concerns regarding micro- and macro-vascular complications, perhaps relating to intravascular thromboses or endothelial dysfunctions [31, 32]. Regarding cardiovascular complications, acute cardiac injury (7~17%), shock (8–7%), septic shock (20%), arrhythmia (16.7%) and heart failure (23%) were reported in hospitalised patients [27–30]. There are few case reports of myopericarditis with cardiac tamponade and pericardial effusion [33, 34]. Ruan et al. reported up to 7% of patients die of fulminant myocarditis and this may be a contributing factor in up to 33% of deaths [35].

Thus, the respiratory viral outbreaks may lead to significant cardiopulmonary failure that is refractory to conventional medical management. During a pandemic, carefully selected patients may be rescued with ECMO, as it warrants excess amounts of limited assets—personnel. Recently published Extracorporeal Life Support Organization (ELSO) COVID-19 guidelines provide recommendations for ECMO use in this setting [36]. The reported complications in COVID-19 are described in Table 1.

**Table 1:** Reported complications with COVID-19

	Total number of patients	Venovenous ECMO %	Pulmonary complications	Cardiovascular complications	Other complications
Huang C [27]	41 hospitalised	NA	ARDS (29%)	Acute cardiac injury (12%)^a^ Shock (7%)	AKI (7%) Secondary infection (10%)
Wang D [28]	138 hospitalised	NA	ARDS (19.6%)	Shock (8.7%), Acute cardiac injury (7.2%), Arrhythmia (16.7%)	AKI (3.6%)
Yang X [29]	52 ICU admitted	NA	ARDS (67%) Hospital acquired pneumonia (11.5%) Pneumothorax (2%)	Cardiac injury (23%)	AKI (29%) Liver dysfunction (29%) Hyperglycaemia (35%) GI haemorrhage (4%) Bacteremia (2%) Urinary tract infection (2%)
Zhou F [30]	191 hospitalised	NA	Respiratory failure (54%) ARDS (31%)	Heart failure (23%) Acute cardiac injury (17%) Septic shock (20%)	Sepsis (59%) Coagulopathy (19%) Acute kidney injury (15%) Secondary infection (15%) Hypoproteinemia (12%) Acidosis (9%)
Varga Z [32]	3 cases	No ECMO	Respiratory failure (3)	Endothelitis in organ vessels (3) Myocardial infarction (1) Reduced LV EF and circulatory collapse (1)	Mesenteric ischemia (2) Multiorgan failure (1)
Xie Y [31]	2 cases	No ECMO	Pulmonary embolism (2)		
Hua A [33]	1 case	No ECMO		Myopericarditis (1) Cardiac tamponade Pericardial effusion	
Inciardi RM [34]	1 case	No ECMO		Myopericarditis with systolic dysfunction (1)	

*AKI* acute kidney injury, *ARDS* acute respiratory distress syndrome, *ECMO* extracorporeal membrane oxygenation, *GI* gastrointestinal, *NA* not applicable

^a^Defined as blood levels of hypersensitive troponin I above the 99th percentile upper reference limit (> 28 pg/mL) or new abnormalities shown on electrocardiography and echocardiography

## ECMO use in recent viral outbreaks

### H1N1

The spring of 2009 in Mexico saw the nascence of the first pandemic of the twenty-first century, the influenza A, H1N1 [37]. This H1N1 virus initially spread through North America, but eventually caused a global pandemic that lasted beyond the usual influenza season in the Northern Hemisphere [38, 39].

Eight studies that reported ECMO use during H1N1 are summarised in Table 2. H1N1-induced ARDS in 2009 resulted in the rapid uptake of ECMO use, and ECMO played an evolving role in critically ill patients [40, 48]. Pham et al. have reported factors associated with death in 123 ECMO treated patients for H1N1-induced ARDS [45]. They concluded that ECMO initiation facilitated the use of ultra-protective ventilation strategy which minimised the alveolar plateau pressure and subsequent pulmonary damage. It was concluded that this minimisation of lung injury was associated with improved outcome compared to conventionally treated patients. No difference in mortality was observed between patients treated with ECMO versus conventional management; however, only 50% of ECMO patients were successfully matched. A specific subgroup of young patients on ECMO with more favourable outcome remained unmatched. The putative benefits of ECMO are still unproven as the improved outcomes may be caused by patient selection. Davies et al. reported the outcomes of 61 patients with H1N1-associated respiratory failure who were supported with ECMO. The mortality rate was 21% in the ECMO group compared to those with conventional treatment, highlighting the promising role of ECMO in future outbreaks causing severe respiratory illness [40]. Although a systematic review to inform decisions concerning the use of ECMO in acute respiratory failure during H1N1 pandemic was published, there was insufficient evidence to strongly recommend use of ECMO for patients with H1N1-induced acute respiratory failure [48]. However, it highlighted that in selected patients, ECMO was associated with improved outcome.

**Table 2:** Demographic data, the patient characteristics and ECMO data of 8 multicentre studies with H1N1 outbreak (2009–2010)

Study group	Data collection/population	ECMO pts./total H1N1 pts.	Age of ECMO pts. (years)	PaO2/FIO2^**a**^ (mmHg)	MV duration^**a**^ (days)	ECMO duration (days)	Discharged alive^**b**^, ***n*** (%)
ANZ ECMO Influenza Investigator [40]	Retrospective/15 ICUs	68/194	34.4 (26.6–43.1)	56 (48–63)	NA	10 (7–15)	32 (47.1%)
UK ERP with SwiFT study [41]	Prospective/4 centres	75^c^	36.5 ± 11.4	54.9 ± 14.3	4.4 ± 3.7	NA	57 (76%)
Italian ECMO network [42]	Prospective/14 ICUs	60/153	39 (32–46)	63.3 (56–79)	2 (1–5)	10 (7–17)	41 (68.3%)
Australian ERP [43]	Retrospective	38	NA	63	NA	NA	33 (86.8%)
Japanese Society [44]	Retrospective/12 ICUs	14	54	50 (40–55)	5 (0.8–8.5)	8.5 (4.0–10.8)	5 (35.73%)
REVA Research Network in France [45]	Prospective/114 ICUs	123	42 ± 13	63 ± 21	2 (1–5)	9.8	79 (64.2%)
Germany ARDS network [46]	Retrospective/40 centres	61/116	42 (39–45)^d^	87 (74–101)^d^	NA	NA	28 (45.9%)
Italian ECMO network [47]	Prospective/14 centres	60	39.7 ± 12	NA	NA	NA	41 (68.3%)

Mean ± SD or median (interquartile range)

*ANZ* Australia and New-Zealand, *ECMO* extracorporeal membrane oxygenation, *ERP* ECMO Retrieval Program, *ICU* intensive care unit, *MV* mechanical ventilation, *NA* not applicable, *pts*. patients, *SwiFT* Swine Flu Triage

^a^Data before ECMO support

^b^Discharged alive of patients who underwent ECMO support

^c^Matched pairs among total 80 ECMO referred patients

^d^Mean values (95% confidence interval)

### Middle East respiratory syndrome

Another coronavirus, namely the MERS-CoV, originated from Saudi Arabia in 2012 and named Middle East respiratory syndrome (MERS). It resulted in 2494 laboratory-confirmed cases predominantly within the Arabian Peninsula [49, 50]. As of November 2019, 851 (34%) confirmed MERS-CoV infections resulted in death. The largest epidemic outbreak outside Saudi Arabia occurred in South Korea in 2015 [51].

Similar to H1N1-induced ARDS, patients with MERS received lung-protective mechanical ventilation and application of early prone positioning with neuromuscular blockade for patients with moderate to severe ARDS (PaO2:FiO2 < 150 mmHg) [52]. Approximately 6% of patients were reported to receive ECMO support as they were unresponsive to conventional treatment [13]. Alshahrani et al. conducted a retrospective chart review on 35 MERS-CoV patients with refractory respiratory failure [14]. Of these, 17 received ECMO and had a lower in-hospital mortality rate than those who received conventional oxygen therapy. We have summarised 6 studies regarding study populations during MERS and ECMO data in Table 3, although we found limited data regarding ECMO use details during MERS outbreak.

**Table 3:** Demographic data, the patient characteristics and ECMO data of 6 included studies with MERS outbreak (2012–2015)

First author	Country	Study design	Study population	ECMO pts./total pts.	Age of ECMO pts. (years)	PaO2/FIO2^**a**^ (mmHg)	MV duration^**a**^ (days)	ECMO duration (days)	Discharged alive^b^, ***n*** (%)
Choi WS [10]	South Korea	Retrospective/multicentre	Ward and ICU	13/186	NA	NA	NA	NA	8 (61.5%)
Rhee JY [11]		Case review/single centre	Ward and ICU	1/5	35	53	0 (4 h)	6	0
Al-Dorzi HM [12]	Saudi Arabia	Prospective/single centre	HCW in ICU	1/8	NA	NA	NA	15	0
Arabi YM [13]		Retrospective/multicentre	ICU	19/330	NA	NA	NA	NA	6 (31.6%)
Alshahrani MS [14]		Retrospective/multicentre	ICU	17/35	45.5 (28.5–58.5)	NA	NA	NA	6 (35.3%)
Shalhoub S [15]		Retrospective/multicentre	HCW in ward and ICU	9/32	NA	NA	NA	NA	4 (44.4%)

Mean ± SD or median (interquartile range)

*HCW* healthcare worker, *ICU* intensive care unit admission, *MV* mechanical ventilation, *NA* not applicable, *pts.* patients

^a^Data before ECMO support

^b^Discharged alive of patients who underwent ECMO support

## ECMO use in ongoing viral outbreak: COVID-19

ECMO may be considered in patients who develop severe cardiopulmonary failure due to COVID-19 which is refractory to optimal mechanical ventilation and other medical therapies [21]. We have summarised data from recently published clinical reports, ELSO registry report and EuroELSO weekly survey in Table 4 to highlight ECMO use during the COVID-19 pandemic.

**Table 4:** Demographic data, the patient characteristics and ECMO data of 3 included studies with COVID-19 outbreak (2019–2020)

First author	Published date/country	Study design	Study population	ECMO pts./total pts.	Age of ECMO pts. (years)	PaO2/FIO2^a^ (mmHg)	MV duration^a^ (day)	ECMO duration (days)	Discharged Alive^b^, ***n*** (%)
Huang C [27]	January 24, 2020/Wuhan, China	Prospective/single centre	Ward and ICU	2 /41	NA	NA	NA	NA	NA
Chen N [53]	January 30, 2020/Wuhan, China	Retrospective/single centre	Ward and ICU	3/99	NA	NA	NA	NA	NA
Wang D [28]	February 07, 2020/Wuhan, China	Retrospective/single centre	Ward and ICU	4 /138	NA	NA	NA	NA	NA
Yang X [29]	February 21, 2020/Wuhan, China	Retrospective/single centre	ICU	6 /52	NA	NA	NA	NA	1 (16.7%)
Guan W [54]	February 28, 2020/China	Prospective/multicentre	Ward and ICU	5/1099	NA	NA	NA	NA	NA
Zhou F [30]	March 9, 2020/Wuhan, China	Retrospective/multicentre	Ward and ICU	3/191	NA	NA	NA	NA	0/3 (0%)
Li X [55]	March 30, 2020/Shanghai, China	Retrospective/multicentre	ICU	8/16	64.3 ± 17.6	66.1 ± 7.8	9.7 ± 5.7	27.1 ± 17.7	3/7^f^
Chen R [56]	April 11, 2020/China	Retrospective/multicentre	Ward and ICU 575 hospitals	171/1590	NA	NA	NA	NA	NA
ELSO registry [57]	April 22, 2020/ELSO centres	–	ECMO	487	49 (41–56)	75 (62–100)^c^	90 (34–135)	190 (118–280)^d^	36/90 (40%)^e^
EuroELSO survey [58]	April 18, 2020/19 countries	–	ECMO	820	52.4	NA	NA	NA	NA^f^

Mean ± SD or median (range)

*ICU* intensive care unit admission, *MV* mechanical ventilation, *NA* not applicable, *pts.* patients

^a^Data before ECMO support

^b^Discharged alive of patients who underwent ECMO support

^c^For 332 cases with data available among total 487 cases

^d^For 200 cases that have completed their ECMO run

^e^For only 109 cases those cases discharged alive/dead

^f^423 cases: ongoing, 217 cases: weaned, 189 cases: withdrawal for death

During the early outbreak of COVID-19 in China, ECMO was employed for those unresponsive to conventional treatment. Initial reports suggested that ECMO has been used in approximately 3% of severe cases with restoration of adequate oxygenation [28]. Wang and colleagues described clinical characteristics of 138 hospitalised patients during very early stage of outbreak in Wuhan, China [28] and reported 36 intensive care unit (ICU) admitted patients. Among these, 17 (47%) required mechanical ventilation and 4 (11.1%) required support of ECMO. As of February 3, the overall mortality was 4.3%. There were more nationwide reports from China, and Chen et al. [56] reported 1590 hospitalised patients and 171 ECMO patients but no specific data of ECMO patients were reported yet. Li et al. [55] have reported 16 ICU patients with 8 ECMO patients. Among those 8 patients, 3 patients survived to discharge, 4 died and 1 was still on ECMO.

Additionally, ELSO registry dashboard [57] provides live updates of ECMO use for COVID-19 cases on ECMO (Table 4). As of April 22, the suspected or confirmed cases were 487. Whilst 288 patients (59%) are still on ECMO, among 90 patients who discharged, 36 patients (40%) survived to discharge. ECMO support type was mostly respiratory (95%), and ECMO mode was mostly V-V (91%). Furthermore, in 4% of patients, ECMO was provided via V-A mode for cardiac and extracorporeal cardiopulmonary resuscitation and 3% had conversion. The EuroELSO survey [58] has now reported ECMO use in over 800 patients as of April 18. Whilst V-V ECMO being the predominant modality used, 423 patients are still on ECMO, 217 patients were weaned from ECMO and 189 ECMO were discontinued due to patients’ death. Further data on patient demography, clinical management aspects and outcomes are awaited. Organisations such as the International ECMO Network (www.internationalecmonet.org) will play a significant role in delivering high-quality research in ECMO.

As the COVID-19 pandemic grows, it is essential that we characterise the pathophysiology in those critically ill patients to guide management and optimise outcome. To assist in obtaining as much clinical data as possible from all ICU patients admitted with COVID-19, the COVID-19 Critical Care Consortium Registry was formed in mid-January 2020 to facilitate data collection, decision support mechanisms through artificial intelligence and a vehicle for future studies regarding ventilation and treatments (ref, unpublished data). Since its initiation to end of March, more than 300 hospitals from 6 continents are participating to characterise critically ill patients and ultimately to reduce their global burden of this disease.

## Conclusions

The experience from previous pandemics has provided preliminary guidance for ECMO use in the current pandemic. The COVID-19 pandemic is unfolding at a time where the better systems for ECMO provision are developed. ECMO is now a well-organised service in many parts of the world; however, inequality remains in terms of access to ECMO. ECMO may not be a therapy that can be extensively used in such pandemic given the resource constraints and availability issues; a responsible use in selected patients is recommended. Although ECMO has a role in critically ill patients, there is currently inadequate data to determine the efficacy, optimal patient selection and management on ECMO. It is essential that we learn and understand throughout the current pandemic, in order determine the risk-benefit ratio of ECMO in COVID-19.

## Data Availability

Not applicable

## Abbreviations

ARDS: Acute respiratory distress syndrome
CESAR: Conventional Ventilation or ECMO for Severe Adult Respiratory failure
COVID-19: Coronavirus disease 2019
ECMO: Extracorporeal membrane oxygenation
ELSO: Extracorporeal Life Support Organization
FIO2: Fraction of inspired oxygen
ICU: Intensive care unit
MERS: Middle East respiratory syndrome
PaO2: Partial pressure of oxygen in arterial blood
SARS: Severe acute respiratory syndrome
SARS-CoV: Severe acute respiratory syndrome coronavirus
SARS-CoV-2: Severe acute respiratory syndrome coronavirus 2
WHO: World Health Organization
